# Post-Traumatic Cerebral Venous Sinus Thrombosis (PtCVST) Resulting in Increased Intracranial Pressure during Early Post-Traumatic Brain Injury Period: Case Report and Narrative Literature Review

**DOI:** 10.3390/healthcare12171743

**Published:** 2024-09-01

**Authors:** Athanasios Theofanopoulos, Athanasia Proklou, Marianna Miliaraki, Ioannis Konstantinou, Konstantinos Ntotsikas, Nikolaos Moustakis, Sofia Lazarioti, Eleftherios Papadakis, George Kypraios, Georgios Angelidis, Georgia Vaki, Eumorfia Kondili, Christos Tsitsipanis

**Affiliations:** 1 Neurosurgery Department, University Hospital of Heraklion, School of Medicine, University of Crete, 71003 Heraklion, Crete, Greecemoustakisnik00@gmail.com (N.M.); xtsitsipanis@gmail.com (C.T.); 2 Intensive Care Unit, University Hospital of Heraklion, School of Medicine, University of Crete, 71003 Heraklion, Crete, Greece; proklath@gmail.com (A.P.); kondylie@uoc.gr (E.K.); 3 Pediatric Intensive Care Unit, University Hospital of Heraklion, School of Medicine, University of Crete, 71003 Heraklion, Crete, Greece; 4 School of Medicine, University of Crete, 71003 Heraklion, Crete, Greece

**Keywords:** traumatic brain injury, cerebral venous thrombosis, post-traumatic, intracranial hypertension, clinical and imaging mismatch

## Abstract

Post-traumatic cerebral venous sinus thrombosis (ptCVST) often remains underdiagnosed due to the non-specific nature of clinical signs, commonly mimicking severe traumatic brain injury (TBI) manifestations. Early recognition of this rare and potentially life-threatening complication is crucial for the effective management of severe TBI patients in Intensive Care. The present study reports the case of a 66-year-old male who was transferred to the emergency department due to moderate TBI. Initial emergency brain computed tomography (CT) scans revealed certain traumatic lesions, not necessitating any urgent neurosurgical intervention. During his stay in an Intensive Care Unit (ICU), multiple transient episodes of intracranial pressure (ICP) values were managed conservatively, and through placement of an external ventricular drain. Following a series of CT scans, there was a continuous improvement of the initial traumatic hemorrhagic findings despite his worsening clinical condition. This paradox raised suspicion for ptCVST, and a brain CT venography (CTV) was carried out, which showed venous sinus thrombosis close to a concomitant skull fracture. Therapeutic anticoagulant treatment was administered. The patient was discharged with an excellent neurological status. To date, there are no clearly defined guidelines for medical and/or surgical management of patients presenting with ptCVST. Therapy is mainly based on intracranial hypertension control and the maintenance of normal cerebral perfusion pressure (CCP) in the ICU. The mismatch between clinical and imaging findings in patients with TBI and certain risk factors raises the suspicion of ptCVST.

## 1. Introduction

Post-traumatic cerebral venous sinus thrombosis (ptCVST) often remains underdiagnosed due to its non-specific nature of clinical signs, commonly mimicking severe traumatic brain injury (TBI) manifestations [1,2]. Cerebral venous sinus thrombosis (CVST), whether traumatic or not, constitutes a rare pathological entity, referring to dural venous sinuses clotting and leading to an impaired venous outflow [3,4]. Most reported rates for CVST range from 3–5 per million, while ptCVST is generally reported in 7-35% of TBI cases [1,5]. In ptCVST, trauma-induced venous drainage impairment results in elevated intracranial pressure (ICP) due to cerebral edema, venous infarctions, intraparenchymal hemorrhage, or reduced cerebrospinal fluid reabsorption [6,7]. Among the reported risk factors, the presence of skull fractures, particularly those located close to dural venous sinuses or the jugular bulb, has most commonly been reported [6,7]. Presently, there are no clearly defined guidelines on the medical and/or surgical management of patients presenting with ptCVST [2,8]. Therapy is mainly based on controlling ICP and maintaining normal cerebral perfusion pressure (CPP) [2,8]. The current literature estimates mortality at around 4–8% [3,9]. The mismatch between clinical and imaging findings in patients with TBI and certain risk factors need to raise suspicion of ptCVST. Early recognition of this rare and potentially life-threatening complication is crucial for the effective management of severe TBI patients. Therefore, the purpose of the present case presentation and narrative literature review is to address this important issue for TBI patients and to narratively integrate the various perspectives regarding ptCVST, along with currently available treatment approaches.

## 2. Case Presentation

**Figure 1:**
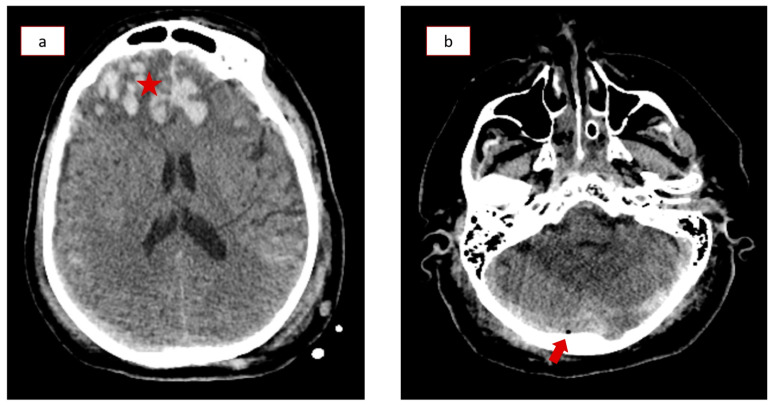
Computed Tomography (CT) scanning upon admission of the patient. (**a**) Hyperdense lesions representing subarachnoid hemorrhage and bilateral cerebral contusions (asterisk). (**b**) Small intracranial collection of air below the occipital bone (arrow).

This study’s case presentation concerns a 66-year-old male patient with an unremarkable past medical history who suffered a moderate traumatic brain injury (TBI) following a 4 m fall. He was immediately transferred to the nearest regional emergency department with an initial Glasgow Coma Scale of 10 out of 15 and mild hemodynamic instability. The patient was intubated, and after primary stabilization, he underwent a full-body computed tomography (CT) scan. Brain and cervical spine CT scans showed several cerebral contusions, pneumocephalus, subdural hematomas, subarachnoid hemorrhage, a fracture of the petrous portion of the temporal bone (Figure 1a,b), and an undisplaced fracture of the second cervical vertebra (Category III of Marshall tomographic score; Injury severity score score of 16). The patient was then transferred to a tertiary hospital intensive care unit with neurosurgical support due to the severity of his condition. Initial ICP measurements (Natus Camino intracranial pressure monitoring catheter, Middleton, WI, USA) were less than 6 mmHg. However, he soon developed several episodes of intracranial hypertension, with ICP measurements as high as 30–35 mmHg, and an external ventricular drain had to be inserted to resolve the condition. 

**Figure 2:**
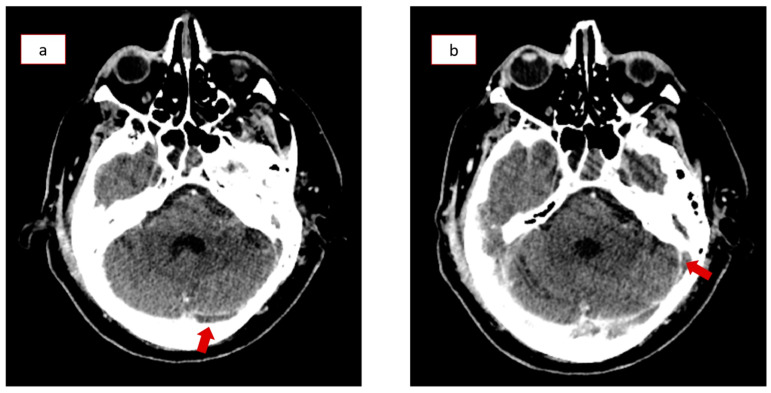
Computed tomography venography (CTV) on the 14th day in the Intensive Care Unit (ICU) revealing thromboses in the (**a**) left transverse and (**b**) sigmoid sinus (red arrows).

During his stay in the ICU, the patient also developed a cerebrospinal fluid (CSF) infection, which was successfully treated with broad-spectrum antibiotics. Despite his worsening clinical condition, the following series of brain CT scans showed continuous improvement of the initial traumatic hemorrhagic findings. The mismatch between his clinical and imaging findings raised suspicion for post-traumatic cerebral venous thrombosis, so the patient underwent a CT venography (CTV) on the 14th day of hospitalization. The CTV revealed left transverse sinus and sigmoid sinus thrombosis (Figure 2a,b), which were treated with anticoagulation therapy (high-dose enoxaparin) according to appropriate protocols (Supplementary Figure S1). The patient underwent a tracheostomy due to prolonged mechanical ventilation, and he was then transferred to the Neurosurgical Department, where a gradual neurological improvement was recorded (GCS of 14/15). He was discharged after thirty-eight days of hospitalization with only minor neurological deficits. The patient presented again with cerebrospinal fluid (CSF) rhinorrhea 36 days after discharge, while new CT scans indicated a bone lesion in the roof of the right ethmoid sinus, necessitating lumbar drain catheter insertion with a continuous flow target of 7–10 mL CSF per hour. He was finally discharged after nine days, and no other complications occurred during his following regular follow-ups.

## 3. Discussion

### 3.1. Epidemiology

CVST, whether post-traumatic or not, constitutes a rare condition, representing 0.5–1% of all strokes, with an incidence of around 2–5 cases per million [10,11,12]. It is more frequent in younger female patients [9]. Common risk factors include thrombophilia, hormonal-related factors (oral contraceptives, pregnancy), systemic autoimmune diseases or head and neck infections, non-cerebral malignancies, or hematological disorders [10,11]. Traumatic brain injury (TBI) is an underdiagnosed cause of CVST (ptCVST) [1,7,11,12,13,14,15]. The prevalence of ptCVST, even in patients with mild TBI and with suspicion of sinus injury, seems to be around 32%, which is primarily related to a fracture adjacent to the sinus involved [6]. Moreover, the superior sagittal sinus is frequently affected, while multiple sinuses seem to be involved in more than 50% of patients presenting with ptCSVT [16]. Recent reports suggest that ptCVST is more common in adults than pediatric populations, although other studies report similar frequencies of this complication for children and adults [7,14,15].

### 3.2. Prognosis

According to a multicenter study involving 624 patients, CVST has a 30-day morbidity rate of around 7.7%, regardless of the cause [4]. Furthermore, the 6-month prognosis is generally poor, with one in five patients experiencing unfavorable outcomes [1]. With regard to CVST, mortality rates stand at approximately 5%, mainly attributable to complications related to comorbidities or CVST-related intracranial hemorrhagic events [12]. On the other hand, ptCVST has been linked to lower mean values of initial Glasgow Coma Scale (GCS) scores, as well as higher scores in the Injury Severity Scoring (ISS) and the Rotterdam CT scoring scales upon admission [4], and has been associated with a mortality rate of less than 10% [4,6]. The prognosis for ptCVST is influenced by various factors, including the patient’s age, the specific sinuses affected by CVST, and the ability of the thrombosed or non-thrombosed sinuses to adapt [2,4,16]. Therefore, it seems that the severity of a ptCVST depends on its localization and the influenced anatomical structures [4], while the discrepancy between the clinical course and brain imaging findings of patients might alert clinicians to rule out ptCVST. Notably, pediatric populations may fare better due to increased plasticity of the sinuses in younger ages [2,16]. 

### 3.3. Signs and Symptoms

Signs and symptoms of ptCVST are highly heterogeneous and can pose a diagnostic challenge [2,11,16]. Patients who are initially asymptomatic might then develop altered mental status, various types of headaches, visual disturbances, seizures, and cranial nerve deficits, indicating ptCVST [11,12]. A change in the type and intensity of headaches could indicate disease progression or complications, such as subarachnoid or intracranial hemorrhage, venous infarction, cerebral edema, or brain herniation [11]. Therefore, close monitoring for level of consciousness alterations or headaches in the post-TBI course is imperative [11,16]. Moreover, intracranial hypertension is the most common clinical symptom of acute CVST, traumatic or not [17]. 

### 3.4. Diagnosis and Radiological Findings

Non-contrast CT (NCCT) is usually the initial diagnostic tool for TBI, but its sensitivity for CVST is only 20–43% [10,18]. Therefore, a normal initial CT scan cannot reliably rule out an active ptCVST [7]. However, certain imaging findings on NCCT, such as skull fractures near a dural sinus, hematoma, intraparenchymal bleeding or hemorrhage of multiple foci, contusions, cerebral edema, pneumocephalus, or signs of venous sinus hyperdensity, such as a cord sign or delta sign, might be suggestive of ptCVST [7,10,11,16]. Thus, early warning clinical symptomatology that is unresponsive to conservative treatment, combined with these imaging findings, should always raise strong suspicion for ptCVST [7,10,11,14] and prompt an emergent contrast-enhanced CTV, which directly detects the venous clot as a filling defect, or a magnetic resonance imaging (MRI)/magnetic resonance venography (MRV) to realize a better description of parenchymal defects as well [19]. Future studies will likely lead to the ultimate guide to patient selection, the best timing, and the appropriate imaging modality for ptCVST diagnosis [7].

Initial imaging studies on asymptomatic patients always carry the risk of not promptly diagnosing a silent ptCVST [20]. However, recent reports indicate that the probability of diagnosing ptCVST increases over time, even 30 days after trauma [7]. Antithrombotic treatments could not be used at the early stages of TBI. Therefore, a proactive, preventive strategy involves performing a standard CT-venography (CTV) within 3–7 days after trauma, when specific indications exist [7]. Delayed imaging studies provide the added benefit of reassessing any new, previously undetected hemorrhagic lesions or signs of ptCVST [7,16]. Following these strategies, a patient with mild TBI and negative imaging after 3–7 days seems to be safe to discharge with regular follow-up visits [7].

### 3.5. The Anatomy of the Cerebral Venous System

Cerebral veins lack muscular tissue and valves, thus permitting blood to flow bi-directionally [19,21]. The cerebral venous sinus system is responsible for draining deoxygenated blood from the cranial cavity to the cardiovascular circulation [22]. This system consists of superficial and deep cerebral veins [21,23]. The superficial venous system drains into the superior sagittal sinus and the cortical veins, collecting blood from the cortex and outer white matter, whereas the deep venous system comprises the transverse sinuses, the sigmoid sinuses, the straight sinus, deep cerebral veins, and the subependymal and medullary veins draining deeper layers of the white matter and basal ganglia [21,23]. Both systems mostly drain into internal jugular veins [21,23]. The development of collateral circulation creates multiple anastomoses that connect cortical veins and could potentially explain the favorable prognosis of many cases of CVST [19]. The absence of a smooth muscle layer in cerebral veins allows them to generally remain dilated, making patients susceptible to venous air embolism during neurosurgical interventions [24].

### 3.6. Pathophysiology

The most effective way to understand the clinical entity of CVST is through pathophysiological theories based on Virchow’s triad [7]. First, the endothelium’s architecture disruption leads to thrombogenic subendothelial tissue exposure, inducing a hypercoagulable state [4,12,19]. The hypercoagulation resulting from blunt head trauma has steadily been identified as an important intravascular predisposing factor for ptCVST [4,12,19]. This has been attributed to the hypercoagulable condition caused by the acceleration of thrombin generation and dysfunction of antithrombin or other anticoagulant mechanisms seen in most trauma patients [2,8]. Moreover, ptCVST can be attributed to stasis caused by factors outside the blood vessels, such as the outer pressure caused by a hematoma, seriously impairing cerebral blood flow [16,19]. Compressive skull fractures, epidural hematomas, and neck collars all induce cerebral blood flow restriction [4,19].

Furthermore, ptCVST leads to venous outflow restriction, resulting in hydrostatic pressure increments of ascending veins and capillaries, ultimately leading to cerebral edema, reduced arterial blood flow, venous infarcts, or blood vessel disruptions and intraparenchymal hemorrhage [16,19]. Venus blood stasis can also induce reduced CSF reabsorption from arachnoid corpuscles that protrude into venous sinuses, deteriorating intracranial hypertension [4,17]. Increased venous pressure finally disrupts the blood–brain barrier (BBB), leading to angiogenic edema, while reduced cerebral blood flow accounts for cytotoxic edema [16,19]. The cytotoxic and angiogenic edema might lead to subsequent infarctions or extension of an existing thrombus [16,19]. Recent research suggests that higher magnitude forces of injury result in more severe thromboses and serious brain damage [7].

### 3.7. Risk Factors and Etiology

Venous thromboembolism has been associated with female sex, pregnancy, and the use of oral contraceptives [16,25,26,27]. Other confirmed risk factors are thrombophilia, concomitant neoplasms, infections, postpartum period, systemic diseases, dehydration, intracranial tumors, hypercoagulation, certain medications, trauma, COVID-19 infection, or adenovirus vaccines [1,2,15,16]. However, the cause remains unknown in nearly a third of all cases [15]. Notably, about 20% of CVST cases are associated with thyroid dysfunction [15,28]. The presence of skull fractures or post-traumatic hematomas close to a sinus or the jugular bulb is the predominant risk factor for ptCVST [2,4,15,16]. All types of hematomas are linked to ptCVST, with a recent study demonstrating that nearly all ptCVST cases had subdural, epidural, or both subdural and epidural subarachnoid hemorrhages or contusions [4,16]. Trauma severity also constitutes a serious risk factor for the development of ptCVST, as previously discussed [29]. 

### 3.8. Relative Frequency of Venous Sinuses Involved

The frequency of sinuses involved in ptCVST varies in different studies, with recent research demonstrating that ptCVST most commonly involves the superior sagittal sinus and often affects more than one sinus [4,7]. According to a recent study, ptCVST more often affected the anterior, middle, or posterior sections of the superior sagittal sinus, with the entire sinus involved in a portion of cases [16]. The transverse and sigmoid sinuses, or the jugular bulb, also seem to be commonly involved in ptCVST, especially following fractures of the petrous part of the temporal bone, while other usual locations of ptCVST include the internal jugular vein or the deep veins of the brain [2,4,16].

### 3.9. Non-Surgical Treatment Options

Symptomatic ptCVST, even if accompanied by a secondary hematoma, should be treated according to the American Heart Association, American Stroke Association and European Federation of Neurological Societies guidelines [4,7]. The goal of treatment, besides symptomatic patient relief, should also be prophylaxis against thrombus propagation and complete vessel obstruction, sinus re-tunneling acceleration, the prevention of a new thrombus or embolus formation into the circulation, and intracranial hypertension control [4,7,11]. According to the European Stroke Organization guidelines (2017), low-molecular-weight heparin (LMWH) remains the mainstay of treatment for acute ptCVST [15]. Anticoagulation therapy guidelines for asymptomatic ptCVST have not yet been standardized [1,2,4,7,16]. 

In cases of ptCVST, it is essential to adopt a distinct clinical approach due to the risk of hematoma rebleeding despite the use of prophylactic anticoagulation doses [14,19,30]. The impact of specific anticoagulation strategies on post-traumatic patients remains quite uncertain, and currently, there are no established guidelines for the treatment of ptCVST [31]. It is necessary to assess whether these strategies offer potential benefits by reducing the risk of further thromboembolic events or if they pose a risk of increased rebleeding [7,19]. Evidence indicates that healthcare providers exhibit hesitancy in initiating anticoagulation therapy for post-traumatic patients [12]. According to a previous study, neurosurgical patients receiving prophylactic doses of LMWH for thromboembolic prophylaxis may face a non-negligible risk of bleeding complications [14]. Certain reports stated that around 43% of patients with ptCVST exhibited substantial clinical improvement and vascular recanalization without anticoagulation therapy [14,30]. Another study reported that hypercoagulation has a minor effect on TBI patient outcomes, suggesting that LMWH should only be considered in selected cases [1]. However, the results of recent meta-analyses advocate the safety of early anticoagulation therapy for traumatic or non-traumatic CVST, regardless of the patient’s age [9,14,15,30]. The initiation of enoxaparin following TBI does not seem to worsen clinical outcomes [31,32] and is currently considered a safe intervention in cases where there is no suspected expanding hemorrhage [33]. The use of dose-adjusted intravenous heparin for critical TBI patients has been recommended since the anticoagulant effects of heparin rapidly reverse within a few hours of infusion discontinuation [30]. A recent study revealed minimal adverse events associated with therapeutic anticoagulation in pediatric patients with ptCVST [34]. Epidural hemorrhage or infections can mimic ptCVST by compressing and displacing a sinus, and therefore, using multiple contrast-enhanced imaging planes can aid diagnosis and help avoid complications from anticoagulation [35,36]. Finally, a recent study reported that even prophylactic doses of anticoagulation might lead to hemorrhagic complications and suggested preventive measures with a control CT scan being performed within the first 48 h after the initiation of treatment [6]. 

### 3.10. Surgical and Endovascular Treatment

The optimal antithrombotic therapy for ptCVST is a topic of ongoing debate due to the lack of clearly defined guidelines to date [2,16,19]. The primary focus of conservative therapy involves implementing strategies to manage elevated pressure within the skull and restore blood flow to the brain [13,19,24]. Recent reports suggest that mechanical thrombolysis with endovascular interventions could be a promising new treatment option for ptCVST [14,19]. This is especially relevant for patients exhibiting persistent neurological deficits or coma despite receiving antithrombotic therapies and experiencing significant intracranial hemorrhages [1,2,15,16]. Recent studies have emphasized the increasing evidence supporting the safety and effectiveness of combining venous angiogram with intravascular thrombolysis using thrombolytic agents like tissue plasminogen activator (tPA) [14,19]. However, due to the lack of relevant studies, their integration into treatment protocols has yet to be established [2,19]. In cases where patients exhibit persistent signs of intracranial hypertension, surgical decompression may be considered as a rescue therapy [16,19]. Nonetheless, recent research has demonstrated that conservative treatment approaches have produced superior outcomes compared to surgical interventions [11,19]. 

## 4. Conclusions

Post-traumatic cerebral venous sinus thrombosis is an uncommon yet potentially life-threatening complication of traumatic brain injury. The presentation of this clinical entity closely resembles that of a severe traumatic brain injury, making it challenging to differentiate. As a result, it is frequently unrecognized. The present research emphasizes the significance of comprehensive planning for contrast-enhanced CT or MRI scans, particularly for TBI patients exhibiting warning clinical signs and concurrent skull fractures near sinuses. Moreover, it is highlighted that the mismatch between clinical and imaging findings in patients with TBI and certain risk factors should raise suspicion for ptCVST. In addition, medical practitioners must carefully weigh the potential complications and contraindications associated with anticoagulant therapy against the risk of untreated patients experiencing thrombus propagation or fatal cerebral edema. 

The current state of knowledge necessitates additional investigations to effectively integrate treatment recommendations for this contentious subject into standard clinical procedures. Future algorithms for refractory intracranial hypertension might even suggest that ptCVST be addressed early in the course of TBI and effectively treated with anticoagulation therapy so that a possible subsequent patient deterioration or even a salvage decompressive craniectomy can be prevented. This mainly concerns cases with certain radiological signs, such as fractures or hematomas adjacent to a sinus, or stable radiological signs on sequential imaging studies, which do not fully explain a patient’s clinical status. 

## Data Availability

The data presented in the study are all contained within this article.

## Supplementary Materials

The following supporting information can be downloaded at: https://www.mdpi.com/article/10.3390/healthcare12171743/s1, Figure S1. Timeline of the patient clinical course during his stay in ICU. TBI: traumatic brain injury; CT: computed tomography; ICP: intracranial pressure; ABX: antibiotic therapy; EVD: external ventricular device; CLABSI: catheter-related blood-stream infection; VAP: ventilator-associated pneumonia; CNS: cerebral nervous system; ptCVST: post-traumatic cerebral venous sinus thrombosis.
