# Supplementary material for: Post-Traumatic Cerebral Venous Sinus Thrombosis (PtCVST) Resulting in Increased Intracranial Pressure during Early Post-Traumatic Brain Injury Period: Case Report and Narrative Literature Review
Source: Healthcare (Basel). 2024 Sep 1;12(17):1743. doi: 10.3390/healthcare12171743 (PMC11395700; doi:10.3390/healthcare12171743)
Supplement: Supplementary file 1 [file healthcare-12-01743-s001.zip › healthcare-3108210-supplementary.pdf]

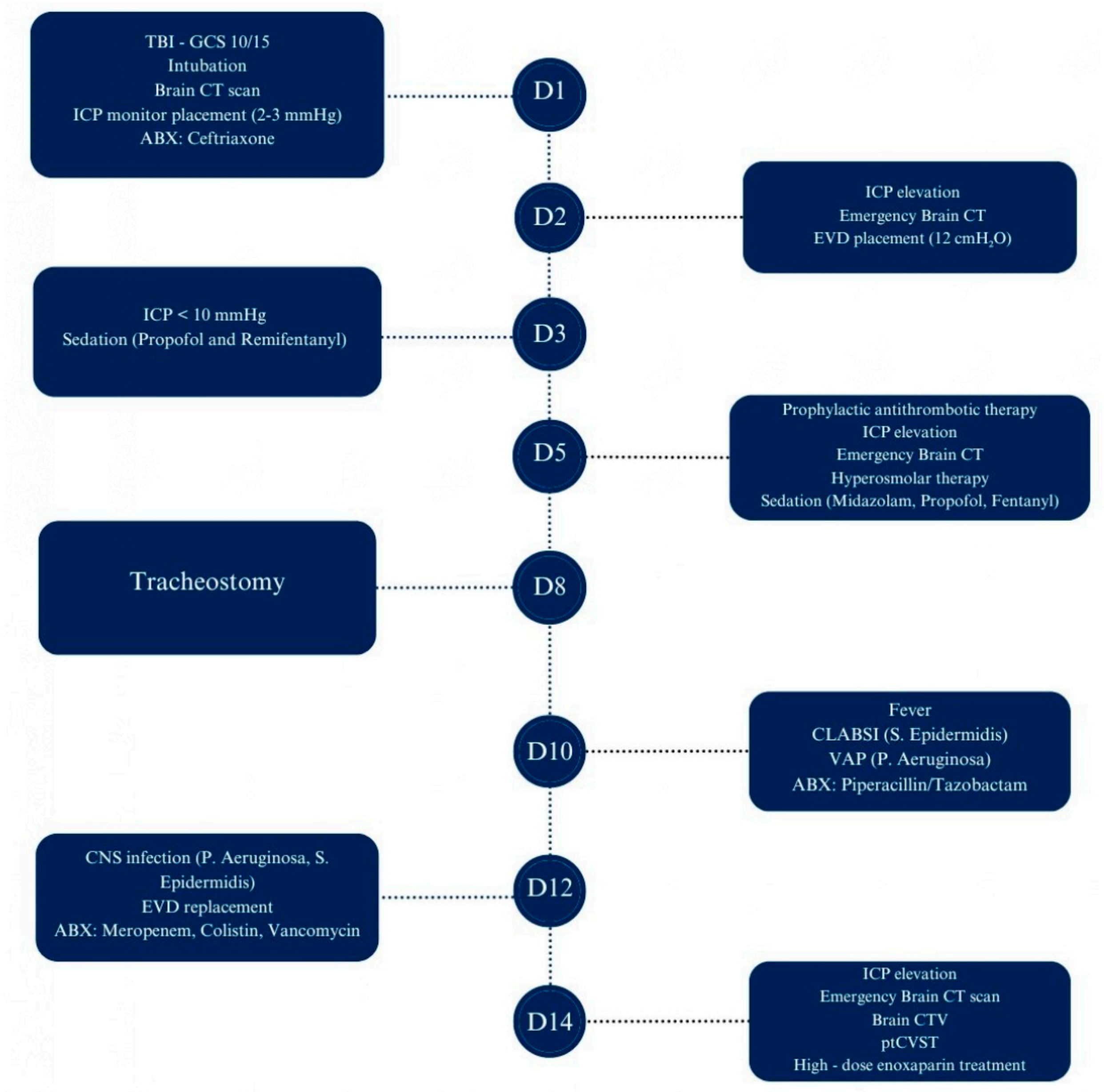

Supplementary Figure S1. Timeline of the patient clinical course during his stay in ICU. TBI: traumatic brain injury; CT: computed tomography; ICP: intracranial pressure; ABX: antibiotic therapy; EVD: external ventricular device; CLABSI: catheter-related blood stream infection; VAP: ventilator-associated pneumonia; CNS: cerebral nervous system; ptCVST: post-traumatic cerebral venous sinus thrombosis.
